# Consequences of *rpoB* mutations missed by the GenoType MTBDR*plus* assay in a programmatic setting in South Africa

**DOI:** 10.4102/ajlm.v12i1.1975

**Published:** 2023-02-06

**Authors:** Nomonde R. Mvelase, Lindiwe P. Cele, Ravesh Singh, Yeshnee Naidoo, Jennifer Giandhari, Eduan Wilkinson, Tulio de Oliveira, Khine Swe Swe-Han, Koleka P. Mlisana

**Affiliations:** 1 Department of Medical Microbiology, KwaZulu-Natal Academic Complex, National Health Laboratory Service, Durban, South Africa; 2 School of Laboratory Medicine and Medical Sciences, College of Health Sciences, University of KwaZulu-Natal, Durban, South Africa; 3 Department of Public Health, Epidemiology and Biostatistics Unit, Sefako Makgatho Health Sciences University, Pretoria, South Africa; 4 KwaZulu-Natal Research Innovation and Sequencing Platform, Nelson R Mandela School of Medicine, University of KwaZulu-Natal, Durban, South Africa; 5 Centre for Epidemic Response and Innovation, School of Data Science and Computational Thinking, Stellenbosch University, Stellenbosch, South Africa; 6 Centre for the AIDS Programme of Research in South Africa, University of KwaZulu-Natal, Durban, South Africa

**Keywords:** tuberculosis, rifampicin resistance, rpoB mutations, MTBDRplus, discordance

## Abstract

**Background:**

Rifampicin resistance missed by commercial rapid molecular assays but detected by phenotypic assays may lead to discordant susceptibility results and affect patient management.

**Objective:**

This study was conducted to evaluate the causes of rifampicin resistance missed by the GenoType MTBDR*plus* and its impact on the programmatic management of tuberculosis in KwaZulu-Natal, South Africa.

**Methods:**

We analysed routine tuberculosis programme data from January 2014 to December 2014 on isolates showing rifampicin susceptibility on the GenoType MTBDR*plus* assay but resistance on the phenotypic agar proportion method. Whole-genome sequencing was performed on a subset of these isolates.

**Results:**

Out of 505 patients with isoniazid mono-resistant tuberculosis on the MTBDR*plus*, 145 (28.7%) isolates showed both isoniazid and rifampicin resistance on the phenotypic assay. The mean time from MTBDR*plus* results to initiation of drug-resistant tuberculosis therapy was 93.7 days. 65.7% of the patients had received previous tuberculosis treatment. The most common mutations detected in the 36 sequenced isolates were I491F (16; 44.4%) and L452P (12; 33.3%). Among the 36 isolates, resistance to other anti-tuberculosis drugs was 69.4% for pyrazinamide, 83.3% for ethambutol, 69.4% for streptomycin, and 50% for ethionamide.

**Conclusion:**

Missed rifampicin resistance was mostly due to the I491F mutation located outside the MTBDR*plus* detection area and the L452P mutation, which was not included in the initial version 2 of the MTBDR*plus*. This led to substantial delays in the initiation of appropriate therapy. The previous tuberculosis treatment history and the high level of resistance to other anti-tuberculosis drugs suggest an accumulation of resistance.

## Introduction

Access to reliable, rapid and automated nucleic acid amplification tests remains one of the key factors in fulfilling the early tuberculosis diagnosis and universal access to drug susceptibility testing (DST) requirement of the World Health Organization (WHO) End TB Strategy.^1^ The past decade has seen the development of many molecular tests, some of which have been endorsed by the WHO. The GenoType MTBDR*plus* assay was the first to be endorsed by the WHO in 2008, followed by the Xpert MTB/RIF (Xpert) in 2010.^2,3^ Because of its ease of use, the Xpert has been implemented in many settings for the initial diagnosis of tuberculosis and detection of rifampicin resistance. Both the MTBDR*plus* and the Xpert detect rifampicin resistance by identifying mutations in the 81-base pair region of the *rpoB* gene, which spans codon 426–452 in the *Mycobacterium tuberculosis* numbering system and codon 507–533 in the *Escherichia coli* numbering system.^4^ This region is also called the rifampicin resistance-determining region (RRDR), as most of the rifampicin resistance-conferring mutations are found in this region.^5^ Additionally, the MTBDR*plus* also detects resistance against isoniazid by identifying mutations in the *katG* gene and the promoter region of the *inhA* gene.

Due to the limitations of the Xpert and the MTBDR*plus* assays, phenotypic methods remain the gold standard for tuberculosis DST. Both assays demonstrate variable performance in detecting heteroresistance and do not detect *rpoB* gene mutations outside the RRDR.^6^ Until recently, mutations outside the RRDR were believed to only account for less than 5% of overall rifampicin resistance.^7,8^ However, in a national drug resistance survey conducted in Eswatini between 2009 and 2010, 30% of multidrug-resistant tuberculosis (MDR-TB: resistant to rifampicin and isoniazid) isolates carried the I491F *rpoB* gene mutation located outside the RRDR.^9^ This caused concerns, especially in neighbouring countries like South Africa, because while this mutation is rare globally, it might be more common in certain geographical settings. A subsequent study conducted in the northern provinces of South Africa showed that 15% of isoniazid mono-resistant strains carried the I491 mutation, meaning they were MDR-TB strains.^10^ The same study also revealed that strains carrying this mutation may be driving outbreaks of MDR-TB in Eswatini and South Africa.^10^

Rapid molecular tests that only detect mutations in the RRDR may fail to detect rifampicin resistance in patients with tuberculosis caused by strains carrying mutations outside the RRDR, and this may lead to inappropriate management, resulting in resistance selection, accumulation of resistance, treatment failure and increased transmission. In settings where molecular and phenotypic rifampicin DST are performed concurrently, discordant results often occur, especially with liquid culture-based assays.^11,12^ Given the fact that rifampicin is the key determinant of the choice of a treatment regimen, the hesitancy caused by discordant results may also affect the decision to start appropriate treatment in the affected patients. Often, an attempt is made to confirm a discordant result by either repeating the test or using another confirmatory assay (if available), thus causing further delay in initiating appropriate therapy.

Because the KwaZulu-Natal province accounts for almost 30% of South Africa’s drug-resistant tuberculosis (DR-TB) cases, and since Eswatini forms part of its northern border, we conducted this study in KwaZulu-Natal, South Africa, to determine why phenotypically resistant isolates were reported as rifampicin susceptible on the MTBDR*plus*.^13^ Considering the dearth of information on the clinical management of patients with rifampicin-discordant tuberculosis results globally, we also report on the programmatic management of these patients in our setting.

## Methods

### Ethical considerations

Ethics approval was obtained from the University of KwaZulu-Natal Biomedical Research Ethics Council (BE267/18). Individual patient consent was not required as only routine programmatic data was accessed; however, permission was obtained from the provincial Department of Health. For anonymity, patients’ names were only used for data collection and were not used during analysis.

### Study design and setting

The study was conducted in the KwaZulu-Natal province in South Africa. The province has the second-highest population in the country with more than 11 million people. There are 11 districts in the province and one MDR-TB treatment facility per district.

In health facilities in the KwaZulu-Natal province, the initial diagnosis of tuberculosis and rifampicin resistance is routinely done using the Xpert (Xpert MTB/RIF, Cepheid, Sunnyvale, California, United States) in all patients suspected of tuberculosis disease. The Xpert was previously used but was later replaced by its successor, the Xpert MTB/RIF Ultra (Xpert Ultra, Cepheid, Sunnyvale, California, United States), in 2017. For patients with rifampicin-susceptible tuberculosis, no further DST is performed, and they are treated using first-line tuberculosis therapy. In patients with rifampicin-resistant tuberculosis on the Xpert (Ultra), a second sample is taken for culture and DST. Other indications for tuberculosis culture include treatment failure and paucibacillary tuberculosis that shows a negative result on the Xpert (Ultra).

During the study period between January 2014 and December 2014, the automated BACTEC Mycobacteria Growth Indicator Tube 960 system (Becton Dickinson, Sparks, Maryland, United States) was used for *M. tuberculosis* culture, and initial DST was done on all positive cultures using the MTBDR*plus* version 2 assay (Hain Lifescience, Nehren, Germany) to confirm rifampicin resistance and test for isoniazid resistance. The MTBDR*plus* assay uses DNA strip technology where the strip contains both wild-type probes and mutation probes for the commonly occurring mutations (S450L, H455Y, H455D, and D435V for rifampicin). The labelled polymerase chain reaction products from an amplified target are hybridised with specific probes immobilised on a strip (reverse hybridisation). Resistance is reported when there is a lack of binding to the wild-type probe with or without binding to a mutation probe.^14^

Isolates that were resistant to either rifampicin or isoniazid on the MTBDR*plus* assay were further tested for resistance to critical concentrations of isoniazid (0.2 µg/mL), rifampicin (1 µg/mL), ofloxacin (2 µg/mL), streptomycin (2 µg/mL), and kanamycin (5 µg/mL) using the 1% agar proportion method on Middlebrook 7H10 agar (Becton Dickinson, Sparks, Maryland, United States).^15^ The simultaneous performance of molecular and phenotypic rifampicin DST allowed the detection of discordance between these two tests.

### Laboratory analysis

Routine clinical isolates from specimens received at the Inkosi Albert Luthuli Central Hospital laboratory of the KwaZulu-Natal province between January 2014 and December 2014 were used for this study. Isolates were selected if they showed rifampicin susceptibility on the MTBDR*plus* but were rifampicin resistant on the 1% agar proportion method on Middlebrook 7H10 agar at a critical rifampicin concentration of 1 µg/mL. The isolates from 2014 were chosen because simultaneous molecular and phenotypic rifampicin DST was performed during this time but was subsequently stopped. The selected isolates were then stored at –70 °C and later used for this study. Of the isolates that had discordant rifampicin results, 36 were randomly selected for further evaluation using whole-genome sequencing.

#### Whole-genome sequencing

Stored isolates were grown on 7H11 Middlebrook agar for over three weeks. Genomic DNA was extracted from isolates using the Quick-DNA™ Miniprep Kit (Zymo Research, Irvine, California, United States). The concentration of DNA was determined using the Qubit dsDNA HS Assay kit (Invitrogen, Carlsbad, California United States). A minimum of 2 ng/µL DNA was used for library preparation. Libraries were prepared using the Nextera DNA library preparation kit and Nextera CD index kit (Illumina, San Diego, California United States) according to the manufacturer’s protocol. Each library was pooled and diluted to an equimolar concentration of 4 nM followed by denaturation and dilution to the final loading concentration. The library was spiked with 1% PhiX, which serves as an internal control to account for low-diversity libraries, and run on an Illumina MiSeq platform (Illumina, San Diego, California, United States) using the Miseq v2 500 cycle reagent kit (Illumina, San Diego, California, United States). Drug resistance and strain-type profiles were determined using the TBProfiler pipeline (http:\\tbdr.lshtm.ac.uk\).^16^ Mutations were called out at 100× depth of coverage.

### Clinical data

Patients with discordant rifampicin susceptibility results were identified from the laboratory. Further laboratory results (phenotypic DST, HIV status, CD4 count, and viral load results) were obtained from the laboratory information system. Treatment data was obtained from the electronic drug-resistant tuberculosis treatment register of the KwaZulu-Natal provincial Department of Health. Treatment outcomes were defined according to the WHO definitions.^17^

### Data analysis

The data were captured into an Excel file (Microsoft Corp., Redmond, Washington, United States) and cleaned and coded before being imported into STATA version 13 (StataCorp, College Station, Texas, United States) for statistical analysis. Patient names and ages were used to remove duplicate entries. Descriptive analysis was conducted on data for all patients with rifampicin-discordant tuberculosis results, as well as those selected for whole-genome sequencing. Categorical variables such as sex, HIV status, previous tuberculosis treatment, as well as the Xpert, phenotypic DST and MTBDR*plus* results, were presented as proportions and percentages. Continuous variables such as age, CD4 count, and the time taken to treatment initiation were presented as means with standard deviation. A bivariate analysis was conducted using the two-sample t-test to compare the mean time taken to treatment initiation between the Xpert-susceptible and Xpert-resistant results, and a *p*-value of < 0.05 was considered indicative of statistical significance.

## Results

In 2014, out of 12 279 *M. tuberculosis* complex cases detected using the MTBDR*plus* assay, 505 (4.1%) were isoniazid mono-resistant. From the 505 isoniazid mono-resistant cases, 145 (28.7%) were MDR-TB based on the phenotypic 1% agar proportion method (i.e., had discordant rifampicin DST results). The median age of the patients with discordant rifampicin DST results was 33.8 years, and 52.4% were male (Table 1).

**TABLE 1:** Patient characteristics of isolates with *rpoB* gene mutations missed by the Genotype MTBDR*plus* assay at Inkosi Albert Luthuli Central Hospital Laboratory in KwaZulu-Natal, South Africa between January 2014 and December 2014.

Characteristics	Isolates with discordant rifampicin results (*N* = 145)		Isolates selected for WGS (*N* = 36)	
	*N*	%	*N*	%
**Sex**				
Male	76	52.4	20	55.60
Female	69	47.6	16	44.4
**Xpert MTB/RIF results**				
Rifampicin resistant	37	38.1	5	0.25
Rifampicin susceptible	60	61.9	15	0.75
**MTBDR*plus*results**				
Isoniazid resistant	140	96.6	36	100.00
Isoniazid inconclusive	5	3.4	0	0.00
**Isoniazid mutation**				
Both *katG* and *inhA*	63	43.4	13	36.10
Not recorded	11	7.6	4	11.10
*katG* only	58	40	17	47.20
*inhA* only	8	5.5	2	5.60
Isoniazid inconclusive	5	3.4	0	0.00
**Phenotypic DST**				
Rifampicin mono-resistant	1	0.7	0	0.00
MDR-TB	43	29.7	10	27.80
Streptomycin & MDR-TB	79	54.5	18	50.00
Pre-XDR FQ†	10	6.9	4	11.10
Pre-XDR SLID†	5	3.4	1	2.80
XDR-TB†	7	4.8	3	8.30
**HIV status**				
Unknown	17	11.7	5	13.90
Positive	89	61.4	23	63.90
Negative	39	26.9	8	22.20
**Viral load**	80	-	23	-
Detected	33	41.3	12	52.20
Not detected	47	58.7	11	47.80
**Initiated DR-TB treatment (yes)**	108	74.5	24	66.70
**Previous tuberculosis treatment**	108	-	24	-
Yes	71	65.7	19	79.20
No	37	34.3	5	20.80
**Tuberculosis treatment outcome**	108	-	24	-
Cured	54	50.0	13	54.20
Died	21	19.4	3	12.50
Treatment failed	7	6.5	2	8.30
Lost to follow-up	13	12.0	3	12.50
Treatment completed	13	12.0	3	12.50
**Treatment duration in months**	18.4	-	19.7	-

Note: Isolates with discordant rifampicin results – Age: median = 33.0, IQR (Q1–Q3) = 23.3–39.7. CD4 count: mean ± s.d. = 288 ± 222. Isolates selected for WGS – Age: median = 32.5, IQR (Q1–Q3) = 24.5–38.0. CD4 count: mean ± s.d. = 296 ± 230.

WGS, whole-genome sequencing; Xpert, Xpert MTB/RIF; DST, drug susceptibility testing; DR-TB, drug-resistant tuberculosis; MDR-TB, multi-drug-resistant tuberculosis, resistant to isoniazid and rifampicin.

† , WHO DR-TB definitions prior to 2021: XDR-TB: extensively drug-resistant TB: MDR-TB plus resistance to any fluoroquinolone plus resistance to any second-line injectable agent; pre-XDR FQ: MDR-TB plus resistance to any fluoroquinolone; pre-XDR SLID: MDR-TB plus resistance to any second-line injectable agent.

### Microbiology results

Out of the 145 isolates with discordant rifampicin DST results, phenotypic DST showed that 79 (54.5%) were MDR-TB plus streptomycin resistant, 43 (29.7%) were MDR-TB, 10 (6.9%) were MDR-TB plus resistance to a fluoroquinolone, seven (4.8%) were MDR-TB plus resistance to a fluoroquinolone and any second-line injectable agent, five (3.5%) were MDR-TB plus resistance to a second-line injectable agent, and one (0.7%) was rifampicin mono-resistant. Xpert results were available for 97 (66.9%) of the 145 patients. Of these, 37 (38.1%) were rifampicin resistant, and 60 (61.9%) were susceptible.

### Treatment details

Patient records were found for 108 (74.5%) of the 145 isolates on the DR-TB treatment register. Of these, 71 (65.7%) patients had a previous tuberculosis treatment history. Sixty-seven (62.0%) patients had favourable treatment outcomes (cured and treatment completed) and the average treatment duration was 18.4 months.

Seventy-six (70.4%) patients on the DR-TB treatment register had an Xpert result; 34 of these were rifampicin resistant and 42 were rifampicin susceptible (Table 2). The mean time to DR-TB treatment initiation was 32.7 days for patients with rifampicin-resistant Xpert results, and 186.6 days for patients with rifampicin-susceptible Xpert results. The difference in the time to treatment between the two groups was statistically significant (*p* < 0.001).

**TABLE 2:** Time from availability of results to initiation of drug-resistant tuberculosis treatment in patients with *M. tuberculosis* isolates with discordant rifampicin susceptibility results in KwaZulu-Natal, South Africa between January 2014 and December 2014.

Rifampicin susceptibility assay results	Number of patients	Mean time from results availability to initiation of DR-TB treatment (days)
Rifampicin susceptible on Xpert MTB/RIF	42	186.6
Rifampicin resistant on Xpert MTB/RIF	34	32.7
Rifampicin susceptible on MTBDR*plus* assay; results available before treatment	61	93.7
Rifampicin resistant on phenotypic test; results available before treatment	23	19.8

DR-TB, drug-resistant tuberculosis.

When calculating the mean time to results in reference to the MTBDR*plus* assay and phenotypic DST results, the 34 Xpert rifampicin resistant cases were removed. Additionally, in two other patients initiated on treatment, the date of initiation was not recorded. Of the remaining 72 patients, 11 patients started treatment before MTBDR*plus* results became available, while 61 started treatment after a mean of 93.7 days from the availability of results. Similarly, for phenotypic DST, 49 of the 72 patients started treatment prior to the availability of results while 23 started treatment after (mean 19.8 days) results were available.

### Whole-genome sequencing results

**FIGURE 1:**
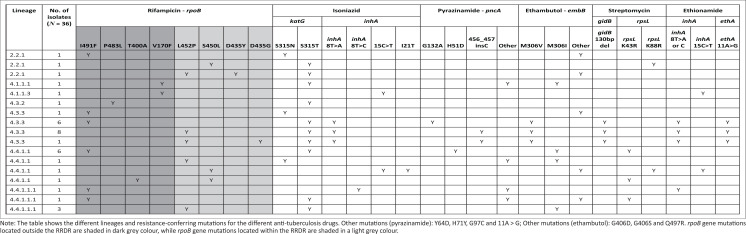
Whole-genome sequencing results of 36 isolates with *rpoB* gene mutations missed by the Genotype MTBDR*plus* assay at Inkosi Albert Luthuli Central Hospital Laboratory in KwaZulu-Natal, South Africa, between January 2014 and December 2014.

Out of the 36 isolates whose whole genomes were sequenced, 19 (52.8%) had single *rpoB* mutations outside the RRDR, namely I491F (16 isolates; 44.4%), V170F (2; 5.6%) and P483L (1; 2.8%) (Figure 1). There were 14 (38.9%) isolates with single *rpoB* mutations located within the RRDR, namely 12 isolates (33.3%) with L452P mutation and two isolates (5.6%) with S450L mutation. The three remaining isolates (8.3%) had double *rpoB* mutations, including one isolate with a combination of the S450L mutation (located within the RRDR) and T400A mutation (located outside the RRDR), and two isolates with double mutations located within the RRDR – one with D435G and L452P mutations and another one with D435Y and L452P mutations.

The most common isoniazid resistance-conferring mutation was the S315T mutation, (29 isolates; 80.6%). This was followed by the *inhA* promoter region mutation T-8A (15 isolates; 41.7%), which was found together with the *katG* mutation in all isolates. Besides resistance to rifampicin and isoniazid, most isolates were also resistant to other first-line drugs, including pyrazinamide (25 isolates; 69.4%), ethambutol (30; 83.3%), and streptomycin (25; 69.4%). Some isolates were also resistant to second-line drugs, including the second-line injectable agents (three isolates; 8.3%), fluoroquinolones (six; 16.7%), and ethionamide (18; 50.0%). One isolate had an insertion in the *mmpR5* gene, which is associated with bedaquiline and clofazimine resistance.

Among the 16 isolates with an I491F *rpoB* mutation, one belonged to sub-lineage 2.2.1, while the remaining 15 isolates belonged to three distinct sub-lineages of lineage 4. Of these 15 isolates, three isolates each had unique mutation patterns, while 12 clustered into two groups based on mutation patterns. One group of six isolates belonging to the 4.4.1.1 sub-lineage carried *katG* S315T, *pncA* H51D, *embB* M306I, and *rpsL* K43R, which confer resistance to isoniazid, pyrazinamide, ethambutol, and streptomycin. Another group of six isolates belonging to sub-lineage 4.3.3 had mutations conferring isoniazid (*inhA* [fabG1 8T > A] plus *katG* S315T), pyrazinamide (*pncA* G132A), ethambutol (*embB* M306V), streptomycin (*gidB* 130bp deletion), and ethionamide (*ethA* 11A > G) resistance.

The 12 isolates carrying a single L452P mutation also belonged to lineage 4. Among the 12 isolates, there were two clusters with common resistance-conferring mutations. The first group consisted of eight isolates belonging to sub-lineage 4.3.3 and had the *inhA* fabG1c.-8T > A plus *katG* S315T isoniazid resistance-conferring mutations, as well as the *pncA* 456_457 C insertion (pyrazinamide resistance), *embB* M306V (ethambutol resistance), *ethA*_11A > G (ethionamide resistance), and the *gidB* 130bp deletion (streptomycin resistance) mutations. The second group consisted of three isolates belonging to sub-lineage 4.4.1.1.1 and carrying *katG* S315T and *embB* M306I mutations for isoniazid and ethambutol resistance.

## Discussion

In this study, almost 29% of the isoniazid mono-resistant tuberculosis cases detected using the MTBDR*plus* assay were MDR-TB cases. This led to significant delays in the initiation of DR-TB treatment. The main cause of rifampicin resistance missed by the MTBDR*plus* assay was the presence of mutations outside the RRDR (mainly I491F), as well as the L452P *rpoB* mutation. Mutations outside the RRDR are not detected by the currently used WHO-endorsed rapid molecular assays, while the L452P mutations were missed by the previous version of the MTBDR*plus* assay. Importantly, isolates carrying these mutations were also resistant to other first-line anti-tuberculosis drugs whose resistance is not routinely tested in tuberculosis patients globally, and the isolates also clustered into distinct groups with unique mutation profiles.

The *rpoB* L452P mutation was left out of the earlier version (version 2, released in 2011) of the MTBDR*plus* assay as it was thought to be clinically insignificant.^18^ This was later corrected in an updated version of the assay launched in 2014.^18,19^ At the time of this study, the older version 2 was still in use, hence the discordant rifampicin results between the MTBDRplus assay and the phenotypic assay in isolates harbouring this mutation. The MTBDR*plus* assay may also miss heteroresistance. One Belgian study from 2019 found that the limit of detection of rifampicin heteroresistance was 5% – 10%.^6^ This may explain the other RRDR mutations missed by the MTBDR*plus* assay in this study. Notably, among isolates that had an Xpert result and had the L452P mutation as detected by whole-genome sequencing, the Xpert assay detected rifampicin resistance.

Mutations outside the RRDR were found in just over half (19/36) of isolates with discordant results that were tested using whole-genome sequencing. If we assume that this proportion is representative of the whole 145 samples with discordant results (i.e. 52.7% of all discordant results are due to mutations outside the RRDR), this will equate to 76 out of 145 discordant isolates. This means that about 15% (76/505) of isoniazid mono-resistant cases had mutations outside the RRDR. This is the same prevalence found by Makhado et al. from Pretoria, South Africa, when they screened isoniazid mono-resistant cases for the I491F mutation in clinical samples collected between 2013 and 2016.^10^ Unlike Makhado et al., who used molecular methods to screen for the I491F mutation, we used the 1% agar proportion method to test for rifampicin resistance missed by the MTBDR*plus* assay. Phenotypic methods, especially liquid-based methods, can fail to detect rifampicin resistance caused by the I491F mutation. In a 2019 study conducted in Belgium by Torrea et al., the agar proportion method detected rifampicin resistance in 75% of isolates with I491F that was missed by the Mycobacteria Growth Indicator Tube DST.^12^ It is therefore likely that the occurrence of these mutations is more frequent than what we found in this study.

**FIGURE 2:**
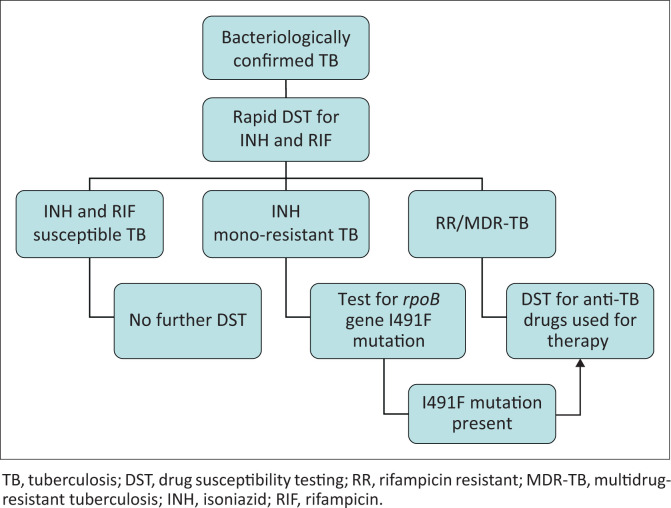
Proposed algorithm for the diagnosis of drug-resistant tuberculosis.

While the overall prevalence of I491F mutation among tuberculosis patients is reportedly low, in patients with isoniazid resistance, the prevalence is high.^9,10,20^ The WHO defines universal access to DST as performing rapid DST for at least rifampicin in all patients with bacteriologically confirmed tuberculosis plus additional DST for at least fluoroquinolones and second-line injectable agents in patients with rifampicin resistance.^1^ The use of Xpert as an entry point to tuberculosis care without investigating isoniazid resistance would prove disastrous for patients infected with *M. tuberculosis* strains that have mutations outside the area of detection and are resistant to all other first-line drugs. Recent studies conducted between 2015 and 2017 have shown that isoniazid resistance generally develops before rifampicin resistance.^21,22^ Notwithstanding the importance of testing for rifampicin resistance, the neglect of isoniazid testing leads to inappropriate therapy, treatment failure and accumulation of resistance in patients with initial isoniazid resistance.^23^ We therefore propose an algorithm to optimise DR-TB detection (Figure 2). We submit that the initial DST should include both isoniazid and rifampicin. Importantly, if resistance is found to any of these two drugs, it should trigger further DST of other first-line and second-line drugs that will be used for treatment. Moreover, an attempt should be made to look for the I491F mutation in isolates from patients with isoniazid mono-resistant tuberculosis as this mutation may be missed by both phenotypic and genotypic DST methods that are routinely used for the detection of rifampicin resistance.

The largest global cluster of extensively drug-resistant tuberculosis that was ever reported was from Tugela Ferry in KwaZulu-Natal in 2005 and it was caused by a strain named F15/LAM4/KZN.^24^ A study conducted by Pillay et al. using *M. tuberculosis* isolates collected between 1994 and 2002 showed how this extensively drug-resistant strain accumulated resistance over time under a tuberculosis programme that lacked appropriate DST.^25^ Patients received inappropriate therapy, thus allowing the selection and spread of resistant strains and leading to treatment failure with dire consequences, especially among patients who were also HIV-positive.^24^ With the current tuberculosis diagnostic algorithm that only tests for rifampicin resistance, we find ourselves in a similar circumstance that calls for swift action if we are to avoid the same unfortunate outcome.

Targeted next-generation sequencing can overcome some of the challenges of rapid molecular assays and phenotypic DST by allowing the rapid detection of *rpoB* mutations outside the RRDR and additional mutations conferring resistance to other anti-tuberculosis drugs, including those that are difficult to test by phenotypic methods (e.g. pyrazinamide). However, the cost, skill levels and expertise required to perform next-generation sequencing and interpret its results remain the prohibiting factors limiting the implementation of this technology, especially in high-burdened, low-resource countries where it is needed the most.^26^ Therefore, in many countries, including South Africa, next-generation sequencing remains confined to the reference and research laboratories.

Although molecular tests have decreased the time to tuberculosis DST results from weeks, using the previous phenotypic tests, to hours and days, discordant results may reverse this benefit. Given the fact that MTBDR*plus* results in this study showed rifampicin-susceptible *M. tuberculosis*, appropriate treatment (DR-TB treatment) could not be initiated until phenotypic DST results showing rifampicin resistance became available. Even so, due to the inferiority of second-line tuberculosis treatment compared to the standard first-line treatment, clinicians may be reluctant to change patient treatment based on a discordant result. The patients in this study often had multiple results, showing that the clinicians sought more evidence before committing patients to DR-TB treatment regimens. As shown in this study, there were significant delays in the initiation of DR-TB treatment, which devalues the benefits of rapid molecular tests.

It was alarming to find such high levels of resistance to other first-line drugs (pyrazinamide and ethambutol), as well as ethionamide and streptomycin. Phenotypic DST for pyrazinamide and ethambutol is not routinely performed in many settings because of poor reproducibility and reliability.^27,28,29^ In the South African setting where Xpert (Ultra) is used for the initial diagnosis of tuberculosis and no further DST is performed for rifampicin-susceptible cases, these patients would be treated with first-line therapy. In fact, given the high number of patients with a previous tuberculosis treatment history and current indications for performing *M. tuberculosis* culture, phenotypic DST was probably performed for these patients because they had already failed tuberculosis therapy. The presence of resistance to streptomycin suggests that these patients may have failed a few rounds of tuberculosis therapy because streptomycin was previously used as part of a standard re-treatment regimen in patients who had failed first-line therapy. In South Africa, this regimen was stopped after the rollout of Xpert, which allowed universal testing of all tuberculosis patients. The rollout of Xpert was completed towards the end of 2013.

Isolates in this study belonged predominantly to lineage 4, which is known to predominate among DR-TB cases in the KwaZulu-Natal province.^30^ Most of the isolates clustered based on the I491F and L452P *rpoB* mutations, with each cluster carrying a unique set of mutations conferring resistance against isoniazid, pyrazinamide, ethambutol, streptomycin, or ethionamide. This suggests that these highly resistant strains may have been spreading undetected in the community. Furthermore, lineage 4.4.1.1 strains with *rpoB* I491F, *katG* S315T, *pncA* H51D, *embB* M306I, and *rpsL* K43R mutations have been linked to an outbreak that originated in Eswatini and later spread to South Africa.^10^

### Limitations

This study reports old data on *M. tuberculosis* isolates from 2014. However, we examined this period because this was when both phenotypic and genotypic rifampicin DST were performed simultaneously in our setting. Moreover, the tuberculosis diagnostic algorithm has not changed since then, although phenotypic rifampicin DST was subsequently stopped. Another limitation of this study was our use of phenotypic DST to select isolates with possible mutations outside the RRDR instead of using molecular screening. This may have underestimated the prevalence of isolates with these mutations as some of them remain susceptible on the phenotypic assay. Due to limited resources, we sequenced only a subset of the isolates with discordant results.

The data from the susceptible tuberculosis treatment register was not available to compare with that on the DR-TB register to determine if patients not listed on the DR-TB register were treated with first-line tuberculosis therapy. Finally, the study was performed in one province of South Africa so the findings may not apply to other regions. Nonetheless, this province has the highest prevalence of DR-TB cases in the country and similar findings have been reported in the northern provinces.

### Conclusion

The presence of highly drug-resistant *M. tuberculosis* strains with mutations missed by the routine rapid molecular assays highlights the need for the revision of the WHO definition of universal access to DST so that tuberculosis diagnostic algorithms include testing for both isoniazid and rifampicin in all patients with bacteriologically confirmed tuberculosis. The recent endorsement of the Xpert MTB/XDR by the WHO for detection of isoniazid, fluoroquinolone and second-line injectable agent resistance in Xpert (Ultra)-confirmed tuberculosis cases provides an opportunity to close the gap in isoniazid testing.^1^ The I491F mutation remains the most commonly detected mutation outside the RRDR and its frequent occurrence in isoniazid-resistant cases calls for its inclusion in assays that detect rifampicin resistance. This codon is not too far away from the RRDR, so current assays can be upgraded to include it to avoid the use of inappropriate therapy, prevent the accumulation of resistance, and reduce community spread.
